# Successful Ostial Stenting in a Patient with a Single Coronary Artery from the Right Sinus of Valsalva: A Case Report

**Published:** 2018-07

**Authors:** Hossein Doustkami, Afshin Habibzadeh

**Affiliations:** 1 *Department of Cardiology, Ardabil University of Medical Sciences, Ardabil, Iran.*; 2 *Department of Internal Medicine, Ardabil University of Medical Sciences, Ardabil, Iran.*

**Keywords:** *Coronary vessels*, *Congenital abnormalities*, *Coronary angiography*, *Percutaneous coronary intervention*, *Stents*

## Abstract

Single coronary arteries (SCAs) constitute a rare coronary anomaly which is usually asymptomatic. However, SCAs may become symptomatic and even cause myocardial ischemia and infarction and as such necessitate proper intervention whether percutaneous or surgical. We describe an 89-year-old woman with an SCA from the right sinus of Valsalva presenting with chest pain and acute myocardial infarction. We succeeded in performing percutaneous coronary intervention and stenting on the ostial lesion of the SCA, and there were no further complications. The patient was discharged 2 days later with no adverse complications.

## Introduction

Coronary artery anomalies are usually detected as incidental findings during coronary angiography and are mostly anomalies of origination and course.^1^ Single coronary arteries (SCAs) constitute a rare anomaly with an incidence rate of 0.024% to 0.066%.^2^ They are mostly asymptomatic but may cause myocardial ischemia, infarction, heart failure, or sudden death.^3^

Here, we report a successful treatment of acute myocardial infarction and stenting of an ostial lesion on an SCA originating from the right sinus of Valsalva. 

## Case Report

**Figure 1:**
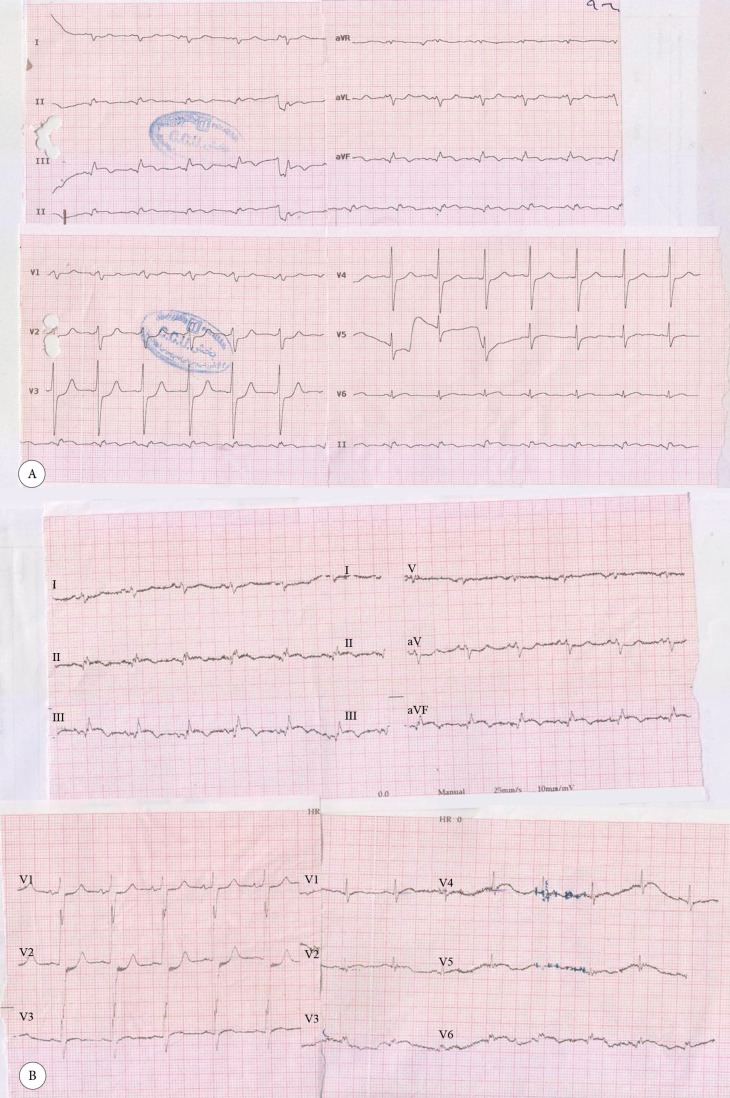
Electrocardiogram of the patient at admission with ST elevation in the inferior, posterior, and lateral leads (A) and after percutaneous coronary intervention with mild ST elevation in the inferior lead and resolved ST/T changes in the posterior and lateral leads (B)

**Figure 2:**
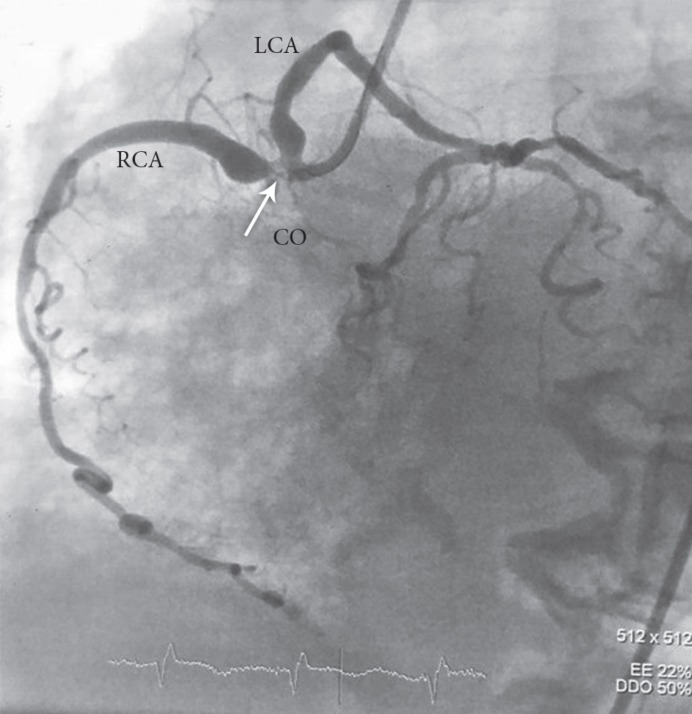
Angiogram of the patient with SCA in the LAO view originating from the right aortic sinus of Valsalva with a significant lesion at the common ostium of the LCA and the RCA (arrow)

SCA, Single coronary artery; LAO, Left anterior oblique; LCA, Left coronary artery; RCA, Right coronary artery; CO, Common ostium

**Figure 3:**
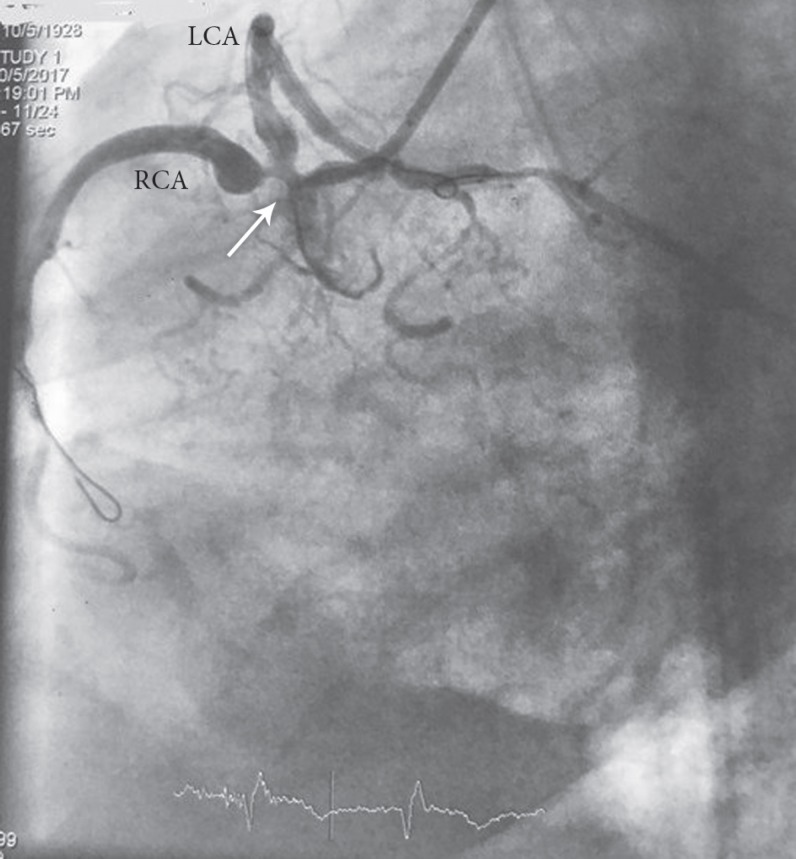
Angioplasty of the patient in the LAO view, showing guide wires in both RCA and LCA (arrow)

LAO, Left anterior oblique; RCA, Right coronary artery; LCA, Left coronary artery

**Figure 4:**
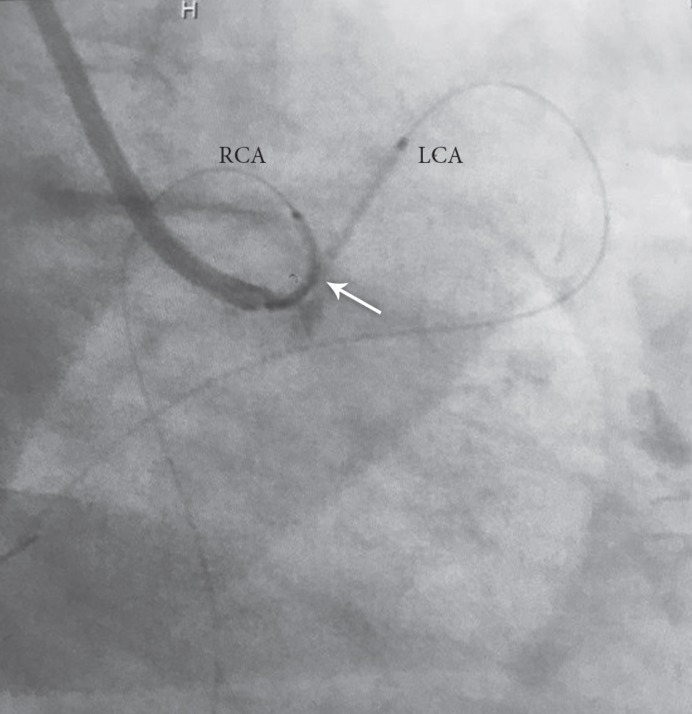
Angioplasty and Y-stenting of the RCA and the LCA (arrow) before stent deployment in the RAO caudal view coronary angiography

RCA, Right coronary artery; LCA, Left coronary artery; RAO, Right anterior oblique

**Figure 5:**
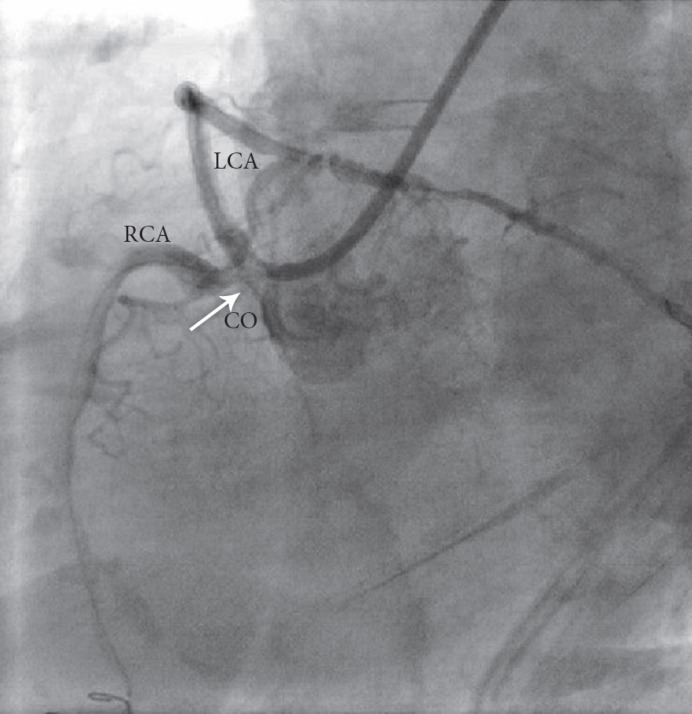
Successful angioplasty with Y-stenting of the RCA and the LCA (arrow) in the LAO view coronary angiography

RCA, Right coronary artery; LCA, Left coronary artery; LAO: Left anterior oblique; CO, Common ostium

An 89-year-old woman with no previous history of cardiac disease presented with chest pain of 3 days’ duration. The patient’s hemodynamics were stable, with a blood pressure of 150/90 mmHg and a heart rate of 78 bpm. The initial electrocardiogram showed ST-segment elevation in the inferior, posterior, and lateral leads (Figures 1A and 1 B). Transthoracic echocardiography showed normal-sized left and right ventricles, severe systolic dysfunction, moderate tricuspid regurgitation, and moderate mitral regurgitation with a left ventricular ejection fraction of 15%. She underwent coronary angiography, which showed anomaly in the shape of an SCA from the right aortic sinus of Valsalva and significant lesions at the common ostium and the proximal part of the right and left systems (Figure 2). Coronary artery bypass graft surgery was recommended to the patient, but she refused. Accordingly, decision was made to perform percutaneous coronary intervention (PCI) with a 7-F right Judkins guiding catheter via the right femoral artery. Both right and left systems were wired with the Balance Middle Weight guide wires, and the lesions were dilated with the simultaneous inflation of two 2.5×15 MINI-TREK dilation balloons (Abbott Vascular, CA, USA). Thereafter, the lesions were Y-stented with two 3.5×115 XIENCE Xpedition stents (Abbott Vascular, CA, USA) and inflated up to 14 mmHg before the procedure was terminated (Figure 3). The procedure was successful, and there were no complications (Figure 4 and Figure 5). The patient was discharged 2 days later with no adverse complications. 

## Discussion

SCAs could be associated with other congenital anomalies, but we found no other anomalies in our patient. We classified the SCA in our patient as R-I according to the criteria by Lipton et al.^4^ This is a very rare subtype with an incidence rate of 0.008%. In the SCA classification, R means that the artery originates from the right side and I indicates that the artery follows the anatomical course of either a right or a left coronary artery.^4^

SCAs may be found incidentally during the evaluation of patients with suspected coronary artery disease, but they can be the cause of myocardial ischemia or infarction. PCI with stenting can be a treatment of choice if the morphology and the route of the anomaly and the coronary artery involved can be accurately determined. Proper evaluation is required for the selection of the most appropriate type of pharmacological, percutaneous, or surgical intervention. In fact, revascularization should be considered only in patients with significant atherosclerotic changes and documented ischemia.^5^

Our patient refused to undergo coronary artery bypass grafting, but we succeeded in performing PCI and stenting. Similarly, Vural and colleagues^6^ reported successful stenting of an RII-type SCA and recommended that PCI be conducted if SCAs become symptomatic. 

## Conclusion

Although coronary anomalies are rare, their clinical significance renders their diagnosis vitally important. PCI should be considered in any patient with stenosis in an anomalous coronary artery. 

## Notes:

***This paper should be cited as: ***Doustkami H, Habibzadeh A. Successful Ostial Stenting in a Patient with a Single Coronary Artery from the Right Sinus of Valsalva: A Case Report. J Teh Univ Heart Ctr 2018;13(3):140-143.
